# Experiences of referral with an obstetric emergency: voices of women admitted at Mbarara Regional Referral Hospital, South Western Uganda

**DOI:** 10.1186/s12884-023-05795-z

**Published:** 2023-07-06

**Authors:** Harriet Nabulo, Helga Gottfredsdottir, Ngonzi Joseph, Dan K. Kaye

**Affiliations:** 1 Department of Nursing, Mbarara University of Science and Technology, P.O.BOX 4010, Mbarara, Uganda; 2 Faculty of Nursing and Midwifery, University of Iceland, Reykjavik, Iceland; 3 The University Hospital of Iceland, Women’s Clinic, Reykjavik, Iceland; 4 Department of Obstetrics and Gynaecology, Mbarara University of Science and Technology, Mbarara, Uganda; 5 Obstetrics/Gynaecology Department, College of Health Sciences, Makerere University, Kampala, Uganda

**Keywords:** Experiences, Obstetric referral, Emergencies, Health care

## Abstract

**Background:**

Life-threatening obstetric complications usually lead to the need for referral and constitute the commonest direct causes of maternal deaths. Urgent management of referrals can potentially lower the maternal mortality rate. We explored the experiences of women referred with obstetric emergencies to Mbarara Regional Referral Hospital (MRRH) in Uganda, in order to identify barriers and facilitating factors.

**Methods:**

This was an exploratory qualitative study. In-depth interviews (IDIs) were conducted with 10 postnatal women and 2 attendants as key informants. We explored health system and client related factors to understand how these could have facilitated or hindered the referral process. Data was analyzed deductively employing the constructs of the Andersen Healthcare Utilization model.

**Results:**

Women experienced transport, care delays and inhumane treatment from health care providers (HCPs). The obstetric indications for referral were severe obstructed labor, ruptured uterus, and transverse lie in advanced labor, eclampsia and retained second twin with intrapartum hemorrhage. The secondary reasons for referral included; non-functional operating theatres due to power outages, unsterilized caesarian section instruments, no blood transfusion services, stock outs of emergency drugs, and absenteeism of HCPs to perform surgery. Four (4) themes emerged; enablers, barriers to referral, poor quality of care and poor health facility organization. Most referring health facilities were within a 30–50 km radius from MRRH. Delays to receive emergency obstetric care (EMOC) led to acquisition of in-hospital complications and eventual prolonged hospitalization. Enablers to referral were social support, financial preparation for birth and birth companion’s knowledge of danger signs.

**Conclusion:**

The experience of obstetric referral for women was largely unpleasant due to delays and poor quality of care which contributed to perinatal mortality and maternal morbidities. Training HCPs in respectful maternity care (RMC) may improve quality of care and foster positive postnatal client experiences. Refresher sessions on obstetric referral procedures for HCPs are suggested. Interventions to improve the functionality of the obstetric referral pathway for rural south-western Uganda should be explored.

**Supplementary Information:**

The online version contains supplementary material available at 10.1186/s12884-023-05795-z.

## Introduction

Motherhood is often a positive and fulfilling experience, though for too many, it is associated with suffering, ill-health and even death for women who fail to access quality basic or comprehensive obstetric care [1]. 40% of pregnant women will have a delivery complication, 15% of these will require comprehensive emergency obstetric care (CEMOC) to manage potentially life- threatening complications to mother.

Obstetric referral is the process of directing or redirecting an obstetric client to an appropriate specialist or agency for definitive treatment [2]. This definitive treatment may be in form of basic emergency obstetric care (BEMOC) or CEMOC provided at different health center (HC) levels within the referral system. Equipment, staff, infrastructure, drugs and supplies are important predictors of the level of preparedness for a health facility to manage obstetric emergencies [3]. The inability of a given HC to perform some or all the signal functions of BEMOC and CEMOC, in the management of obstetric complications warrants referral of the client to a better equipped health center [4]. Referral is necessary because not all the care needed may be available at the lower levels of healthcare [5].

The health care system in Uganda operates on referral basis; national, regional, district referral hospitals, to health center IVs, IIIs, IIs and Is. Health center IIIs offer BEMOC while HC IVs through to national referral hospitals offer both BEMOC and CEMOC services. HC IIs offer only antenatal care, immunization, and outpatient services. The HC I is not a physical structure but is constituted by village health team (VHT) members whose role is identifying women with danger signs and are charged with the function of performing referral [6].

An effective, functional referral system is critical to ensuring access to appropriate and timely emergency obstetric care services which would contribute to reduction in maternal mortality and morbidity [7]. There are disparities in accessing emergency obstetric care between urban and rural populations in Uganda. Rural women often experience delays to make a decision to seek care, delay to reach place of care and delay in receiving appropriate and adequate care [8].

A critical challenge for referral is the inadequate capacity of the health facilities, especially the health center IVs, to handle emergencies such as caesarean sections or blood transfusion [4, 9]. Women need effective clinical care provided by kind and competent health workers who are working within a well-functioning health system. An effective referral system with communication between facility- and community-based care providers and between health and transport workers in case of complications, is important at the time when maternal emergency services are required [10].

In a study assessing effectiveness of maternity health care in Tanzania, it was not clear why late obstetric referrals were persistent [11]. They recommended further qualitative studies employing in-depth interviews about non-compliance to maternal referral so that these could reveal more information on experiences and perceptions of the women and their families on the referral systems, probably digging deeper to explain the mystery behind these persistent late obstetric referrals [12] .

Barriers to implementing referral procedures in the rural Tanzania and South Uganda as reported by health care providers (HCPs) include; having to implement a rigid protocol, difficulties in communicating the indication of referral to clients, inability to complete the referral process for their clients, no communication prior to referral, no feedback from main hospital to lower health facilities, medical supplies, support supervision and harassment by colleagues [13, 14]. Inadequate use of ambulance services, poor management of patients during transit, lack of professional escort, unannounced emergency referrals, lack of adequate feedback, drugs and blood were cited by patients, their attendants and health workers as the main barriers to referral to a referral hospital in Ghana [15].

Data from studies documenting women’s experiences of obstetric referral in south western Uganda is scanty. We explored women’s experiences before and after they arrived at MRRH for care. Our study was informed by the Andersen Healthcare Utilization model which describes how health system organization (external environmental factors including barriers to access), enabling factors and client characteristics (predisposing factors) influence women’s experience and perception of care as they interface with health care facilities. Predisposing factors include a woman’s individual characteristics including her demographic factors that influence what she encounters at referral. The need factors refer to the actual obstetric complications like severe obstructed labor that led to the need for her to be referred. [16].

Some situations around a given woman at the time of referral may facilitate her transfer to hospital (enablers) like having money for transport fare or hinder her ability to transfer timely to the hospital (barriers to referral) like no ambulance vehicle. These barriers to referral could have accounted for delays to decide to seek care, delays to reach emergency care health facilities and delays to receive emergency obstetric care and were secondary indications to referral in addition to the actual primary obstetric indications. Delays lead to late obstetric referral and consequent poor maternal outcomes [17].

## Methods

### Study design

Qualitative descriptive studies are useful in uncovering the full nature of a little-understood phenomenon. They allow a researcher to explore a topic with limited coverage within the literature. Using interviews gives focus to the investigation [18]. This was an exploratory qualitative study that described the experiences of women referred with an obstetric emergency to Mbarara Regional Referral Hospital (MRRH).

### Study country and setting

Uganda has one of the highest maternal mortality rates in the world which stands at 336 deaths per 100,000 live births; this translates into 16 mothers dying per day [19] most of which are from late obstetric referrals.

The study was conducted at MRRH which offers a variety of specialist health care services, including BEMOC and CEMOC. It is the teaching Hospital for Mbarara University of Science & Technology and several health training institutions. It serves a catchment population of about 2 million people from the districts of Buhweju, Bushenyi, Ibanda, Isingiro, Kiruhura, Lyantonde, Mbarara, Ntungamo Mitooma, Rakai, Rubirizi and Sheema. MRRH also receives referrals from other regional referral hospitals; Kabale and Fortportal Regional Referral Hospitals, district hospitals like Itojo and Nyakibale and serves clients from the neighboring countries of Tanzania, Rwanda as well as refugees from the Democratic Republic of Congo and Burundi [20]. Its maternity unit registers about 10,000 births annually; about 20 spontaneous vaginal deliveries, 5 caesarian sections including 10 obstetric referrals daily from lower health units all-over South-Western Uganda. The hospital has a cesarean section rate of 40% and an obstetric referral load from the peripheral health facilities of 1,200 per year. The department of Obstetrics and Gynecology is manned by 15 Obstetricians and 28 midwives and offers a 24 h -service daily [21].

### Study population and sampling strategy

The study was conducted in May 2022. We used the maternity ward admissions’ register which has a column to show referred clients and reason for referral. We confirmed the identity of the referred clients by reading through their case notes to obtain the primary reasons for referral (obstetric indication for referral), secondary reasons for referral (health system barriers to referral) and the referring health center. We checked with the woman to ensure she is one captured in the admissions’ book. We recruited women referred with the following complications: ruptured uterus, eclampsia, and retained second twin with intra-partum hemorrhage, transverse lie in advanced labour and severe form of obstructed labour. These included institutional (health facility) and traditional birth attendant (TBA)/village health team (VHT) member referrals with referral notes or any documented evidence of pre-referral medical management. TBAs and VHTs are community members with no formal midwifery trainin charged with the role of referral.

We selected participants receiving care in the normal postnatal wards and excluded those in intensive care unit and high dependency unit because they were too ill to be interviewed. Participants were purposively selected. In-depth interviews (IDIs) were conducted until saturation at 13 participants (11 women and 2 attendants, a spouse to a woman who got an emotional breakdown and could not continue with the interview and requested her partner to narrate the rest of the referral encounter and a mother to a post eclamptic woman who could not tell some of the events that occurred as she had convulsions- spouse coded R2B and mother R3B). Background information from the participants like age and parity was collected during the interviews and all the eligible participants approached agreed to participate in the study.

We used purposive maximum variation sampling. There was diversity during recruitment of participants in terms of age, marital status, education level, socio economic background, and distance to MRRH, previous referral experience, type of emergency to document varied views, opinions, and experiences. Despite the differences in obstetric indication for referral, saturation was attained when no new information was being given and experiences being shared were becoming similar. Saturation is taken to indicate that, on the basis of the data that have been collected or analyzed, further data collection or analysis are unnecessary [22] since it provides no new ideas.

### Data collection tools

In-depth interviews were chosen because they can be used to explore the views, experiences, beliefs and motivations of individual participants [23]. The interview guide was developed based on the literature reviewed and relevant probes employed to capture client experiences during transfer to MRRH. It had about 15 open-ended questions capturing client background characteristics, experiences, enablers, and barriers to referral. It was piloted at another regional referral hospital on three women referred with severe obstetric complications to ensure reliability of the questions in answering the research questions. Vague questions were removed or rephrased after the pilot study. The interviews started with exchange of greetings and a short preamble about the study purpose. We then proceeded with an open-ended question where respondents described what had happened during transfer to hospital.

### Data collection

Written consent was obtained from participants after thorough explanation of the study purpose. Interviews were conducted after the clients had given birth or their conditions stabilized in a side room to ensure privacy and to facilitate independent responses without external manipulation. Interviews were conducted by the first author, who is a nurse-midwife in the local dialect; *Runyakitara* and lasted between 40 and 50 min. One participant had a stillbirth and another had been referred with eclampsia. Their conditions were sensitive to proceed with the interviews. A spouse and mother to these clients were interviewed as key informants using the same guide to complete their interviews which had been interrupted by an emotional breakdown and non-recall respectively. The interviews were, audio recorded, transcribed from *runyakitara* into English by first author.

### Data analysis

HN read and reread the transcripts, took notes, and made sense of the texts. Latent content analysis as suggested by Lundman [24] was used. First, in-depth interpretation of the content of text through a systematic classification process of coding was conducted, then identifying patterns, condensing data without losing its quality was done. Coding of the data and the analysis were done manually and deductively using a conceptual framework guided by the constructs of the Andersen Healthcare Utilization model. The model focuses on three core factors to explain healthcare utilization : predisposing, enabling and need factors [25]. Predisposing factors are those associated with the individual receiving care, such as the client‘s demographic characteristics, need factors refer to the obstetric complication that led to the need for referral. Enabling factors are those that relate to facilitators to heeding referral advice such as the woman’s birth preparedness and complication readiness plan and availability of a functional ambulance system. The unit of analysis was an individual interview. Quotes that best described the various themes and subthemes and expressed what was frequently said by the different participants were chosen. After several reviews, all the authors agreed to the formulated subthemes and themes.

### Ethical considerations

The study was cleared by the Mbarara University Research Ethics Committee (REC). Administrative clearance to collect data was sought from the Executive Director for Research, Mbarara Regional Referral Hospital (MRRH). The purpose of the study was explained to all the participants, and written informed consent was signed before participation. They gave their information voluntarily and were told that decline to participate did not have any penalties related to access to ongoing or later care.

**Fig. 1:**
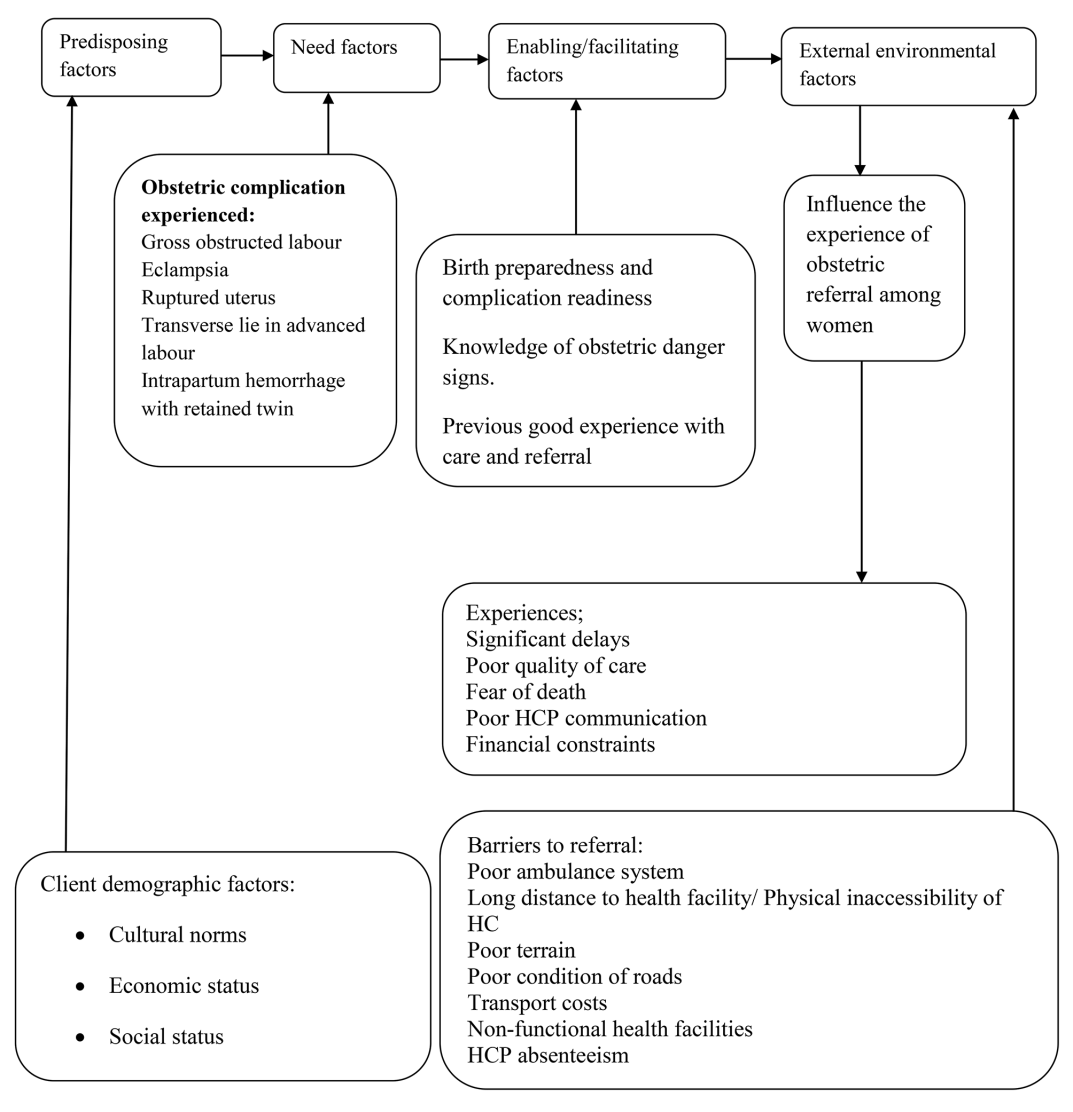
Conceptual framework modified from Andersen’s Health Care Utilization Model [25]

Figure 1 represents how an interplay of client background factors, external environmental factors like poor ambulance system, enablers and barriers to referral all have an influence on woman’s experience of transfer to seek BEMOC or CEMOC.

## Results

### Demographic characteristics of the participants

Participants were mostly married, with low education status and no formal employment. The participants were aged 19 to 38 years of age. We interviewed 11 women with obstetric complications and 2 attendants as key informants. Most participants were referred from a 34–60 Km radius from MRRH. The demographic characteristics of our participants are represented in Table 1.

**Table 1:** Demographic characteristics of the participants [26]

Participant code	age, tribe, parity	marital status	education level	occupation	distance to MRRH
**R1**	22, Munyankole, ,para 1 + 0	married	primary 5	peasant farmer, housewife	over 150 km (moved from 2 HCs before finally reaching MRRH)
**R2A**	28, Munyankole, para 3 + 0	married	Primary 6	farmer, housewife	about 60 km moved from 1 HC to MRRH,
**R3A**	Para 35, 5 + 0, Muhima	married	Primary 7	farmer	
**R4**	23, Munyankole, para1 + 0	married	primary 7	housewife	over 60kms ,transferred from 2 HCs
**R5**	19, Munyankole, para1 + 0	married	senior one	farmer, housewife	about 9 km
**R6**	35,Munyankole, para7 + 0,	married	primary 5	peasant farmer	about 34kms, moved from 1 HC to MRRH
**R7**	27,married, Munyankole,para1 + 0	married	tertiary after senior 4	teller in a SACCO(credit facility)	about 34, moved from 1 HC to MRRH
**R8**	32, Munyankole, para 5 + 0 now, 3 living.	married	primary 7	peasant farmer	about 50kms, moved from 1 HC to MRRH
**R9**	29, Para 4 + 0, Munyankole	married	primary 6	housewife, farmer	about 35 kms
**R10**	27, Munyankole, para3 + 0 (3 sets of twins now)	married	None	peasant farmer, housewife	about 34 kms (Moved from I HC)
**R11**	20, para 1 + 0, Muhororo	married	primary 7	peasant farmer, housewife	over 150 km (Moved from 2 HCs before finally reaching MRRH)
**R2B**	33, male, Muhaya	married		peasant farmer	over 60 kms
			none	farmer	
**R3B**	50, Muhima	widow	none	farmer	over 60kms

**Fig. 2:**
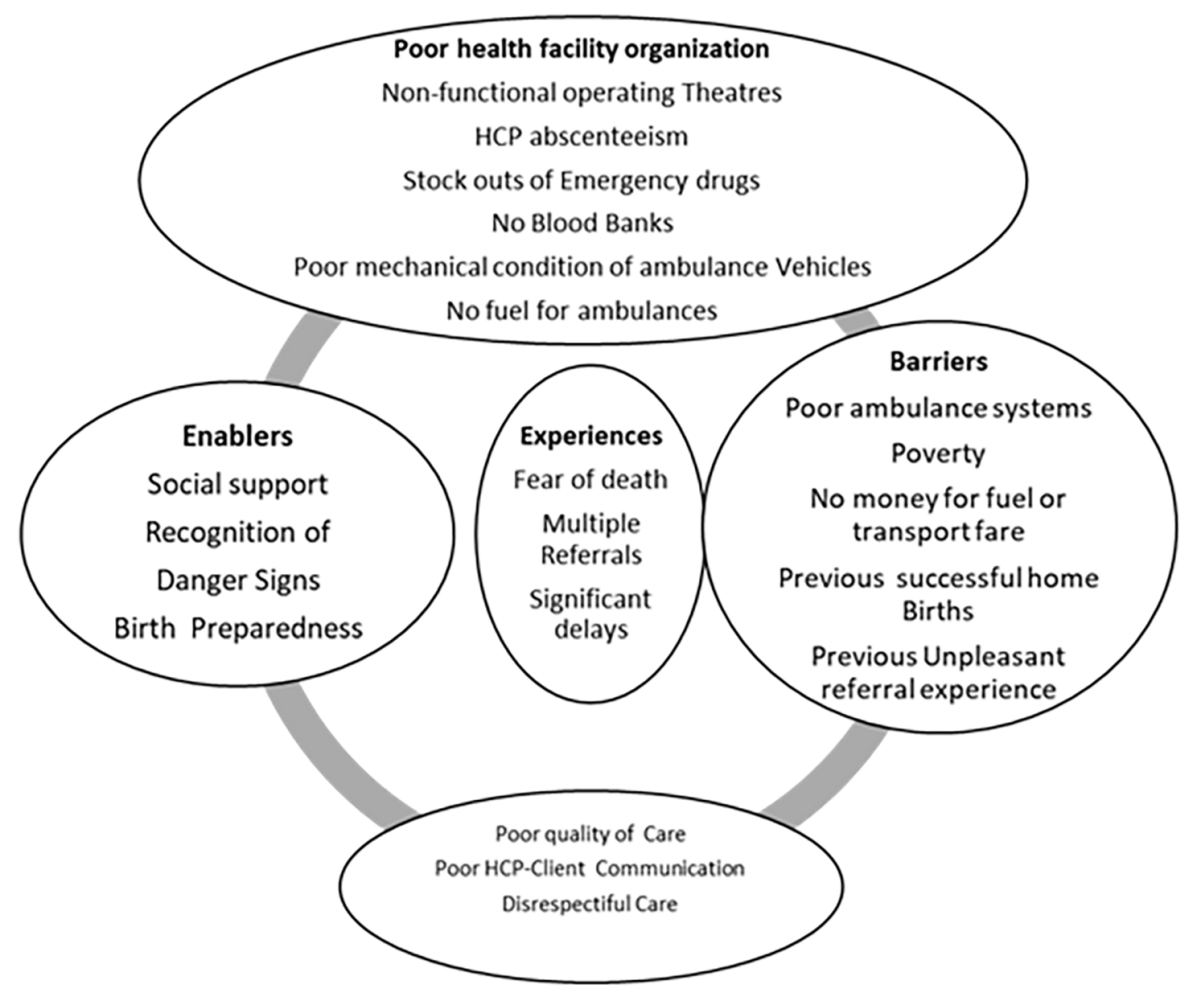
Themes and subthemes; Experiences of referral to MRRH with an obstetric emergency/complication [27]

Women´s experiences are the core of the presented data. These were affected by significant delays, multiple referrals, and fear of death, financial constraints and exhaustion in labor. The findings resulted in 4 themes and 19 sub-themes which we have presented in Fig. 2 below. These experiences were mainly negative.

The themes are; enablers, barriers (to referral), poor quality of care and poor health facility organization.

The theme ‘poor health facility organization’ was coined from the subthemes; HCP absenteeism, non-functional operating theatres, no fuel for ambulance vehicles, poor mechanical condition of ambulance vehicles, stock outs of emergency drug and no blood bank.

The ‘poor quality of care’ theme emerged from the subthemes of poor HCP-client communication, unethical HCP behavior and disrespectful treatment.

The theme ‘enablers to referral’ developed from the subthemes of; knowledge of obstetric danger sign, social support and birth preparedness.

The ‘barriers to referral’ theme is supported by the subthemes: long distance to health facility, poor ambulance system and poverty (no money for transport fare).

### Experiences of referral

The experiences include; significant delays, fear of death and multiple referrals.

#### Significant delays

Participants reported delays before finally reaching MRRH. There were long distances to HCs, multiple referral from HC to HCand interruptions in the journeys during breaks to load and offload other passengers where public transport was used. Transport delays while one had pain was rather exhausting for them. There were delays to receive care and participants who had still births felt that their babies could have been saved by a timely operation.

*“He didn’t have medicine labour pains, so he told me to go to another clinic. At the second clinic the HCP was heading for a burial, so I was taken to a government HC* where *I spent 2 days. They made me to push hard in vain, the baby didn’t come. HCPs didn’t allow me to sleep, they examined me now and again in my private parts and instructed me to continue pushing but I got really tired, the chest was paining. They gave me a drip for more labour pains. i pushed all night until I got exhausted, then I told them to give me a note and send me to a bigger HC. At the 4th HC, the nurse said that since I had already pushed for long, they should take me to Mbarara hospital directly because they hadn’t been with electricity for two days and didn’t have the equipment to use in order to operate upon me.”* R11 para 1 referral with obstructed labour.

*“The baby was to be borne at 1am because that is the time when the baby’s head appeared. In the morning we remained there and the HWs told me that the baby will come out but it didn’t and later they wrote me a letter and we came here.* A*t first when the baby’s head appeared, we were told to look for the baby’s clothes. Later the HW came back and said that time to give birth had not come. I asked him why then he had sent for baby clothes. He said he thought the baby had come but it turned out not. I could also touch and feel the baby’s head down here. I almost lost this baby boy because of overstaying at that health facility. In the theatre here they found my womb torn but it was repaired. I later got lots of pus in my woumb”* R4 a 19-year-old, Para 1 + 0, referred to MRRH with Obstructed labour from a HC III.

*“They took my wife to theatre at 10pm and she was worked on at 2am. They should have worked on her first since we had come in earlier than all those who were worked on first .It is unfair, now we have lost our baby and my wife’s womb”* (R2B husband to R2A, para3 + 0, referral with obstructed labor, found with ruptured uterus at operation).

#### Fear of death

The fear of being the next potential maternal death loomed over the entire maternity wing at a HC IV when a pregnant woman who came in unescorted died shortly after arrival. The woman passed on in full view of the other women in labour. This caused intense fear among them who imagined they could be the next to die. Some worried if they could arrive at MRRH alive.

*“Of course, it was too bad to see a colleague die at the health center you are also attending. It is scary, but since we had labour pains they couldn’t stop, there was no escape for us.”* (R6 referral with obstructed labour about 34kms away).

*“On the way I didn’t even know we would reach here when she is still alive. She would fit almost every 5 minutes. No he drove very fast I actually got worried that she would become worse due to the speed. I think I was confused by her condition to even tell the time correctly.”* (R3B, a mother to a referral with eclampsia).

Following severe obstructed labor, some got severe puerperal sepsis, ruptured uteri and underwent hysterectomy. Others developed vesico-vaginal fistula during the prolonged hospitalization. This morbidity caused the immense grief.

*“The health workers said that my womb was getting rotten, so they removed it’. Imagine this was my first pregnancy so I will never have a child. This urine that is leaking, when will it stop, how will I be able to move among people’*…. said amid sobs. (RI Referral with obstructed labor, who was referred from a HC over 150km away from MRRH.

### Poor quality of care

This theme was coined from the subthemes of disrespectful treatment and poor HCP-client communication.

#### Poor HCP-client communication

Participants reported poor communication between them and the HCPs. The clear reasons for referral to MRRH were not communicated to some of them and while at the HCs, were not always updated on their progress. When they asked, they received cold treatment (rude replies or silence). Money was solicited from some of them, yet they were attending government-owned health facilities where services should be free of charge. They were then sent to MRRH when they failed to pay. Participants felt that the HCPs were non-courteous, with unethical behavior, and this caused dissatisfaction with care.

*“They did not update us on what was happening in theatre so we waited until morning at around 9am they were calling people to receive their babies but I was the last to be called so somehow I thought that since I was the last to be called may be the baby is not well .So they called the lady I was with and told her to bring baby clothes and later they called for baby’s father and so I went, they gave me the dead baby we took her for burial.”(*R2B attending to wife R2A referral with ruptured uterus secondary to obstructed labour, about 60kms away).

#### Disrespectful treatment

Some women experienced disrespectful care from HCPs who were rude and unkind and ended up with adverse pregnancy outcomes, as exemplified by one woman, a referral from a HC about 200kms away due to obstructed labour. She was a primepara, gave birth to still birth and later got sepsis, peritonitis, hysterectomy was done and she later developed foot drop. She also got a burst abdomen and was taken to theatre three times. She developed vesico-vaginal fistula.

“*The nurse said that you know you are poor so where are you going to get the money to take you to a bigger HC. She talked to us rudely. I cried & told them that I won’t go back to the labour room to push. I had pushed for so long. The legs got numb, I was carried into the vehicle & we came here. I hate those HWs at the 1st government HC where I went, they refused to give me a referral letter in time. The person am annoyed with is that nurse. I actually got damaged by too much pushing. I lost my womb. We have so far spent about 700,000 Uganda shillings buying drugs, gloves and food. I really feel bad about myself.’*’R10.

Some HCPs displayed unethical behavior by making women pay for services in government health facilities where user fees were abolished. The participants viewed this as unacceptable as can be seen in one of the excerpts below;

*“If you had delivered normally, you would pay us 80000, but now that you have not given birth, give us 45000 ugx. That is what those HWs did to us. Remember we didn’t have enough money on us…….ya…. Imagine paying in a government- owned health facility. That is what your fellow HWs are capable of doing.*” (R4, primipara, referral with obstructed labour, referred from a HC about 9kms to MRRH).

### Enablers to referral

This theme emerged from the subthemes of social support, birth preparedness and recognition of danger sign.

#### Social support

Participants were able to make it to MRRH owing to the support from family and significant others (birth companions) in form of escort or lending them money. Compassionate car owners reduced on the cost of car hire, transported them for free or on credit, as exemplified by some respondents, who reported social support from community members.

“*The driver allowed to bring us on credit, which is why we never used the HC ambulance as we didn’t have cash to fuel it*.” (R4, referral with obstructed labour, over 60kms moved to MRRH).

“*We didn’t have enough money but the driver was kind and reduced for us on the cost of car hire.”* (R3A, referral with eclampsia, referral from a HC over 34kms away from MRRH).

*“We have a neighbor, an old man; he is a family friend and he has a vehicle, he transported us to Kabuyanda and then then brought us to Mbarara. We help each other in many ways so we didn’t pay him at all.”* (R8, referral with transverse lie, impending rupture of uterus.)

#### Birth preparedness

Birth preparedness aided the referral process for some of the participants. Women reported having saved money during the antenatal period for use if need arose. These savings were later used for car hire. This facilitated them to heed referral advice as captured from the excerpt below;

*“I saved some money earlier during the pregnancy because I knew this was not a simple pregnancy, I didn’t want to start asking for financial help at the last minute with no assurance that I would get it, so I kept some money on me.*” R9, referral with retained second twin).

#### Recognition of danger sign

Some birth companions were knowledgeable about danger signs like difficulty in breathing and when these appeared in their own, they hastened to take them for management at BEMOC centers.

*“My mother says she wondered to herself that if she had left me to spend a night at home, what was pressing me in the chest could spread to the abdomen since I was pregnant, and then kill me. So she rushed to the nearest health center though it also sent us here because there was no medicine to stop my fits”* (R3A, referred with eclampsia.)

### Poor health facility organization

This theme emerged from the subthemes of health care provider absenteeism, recognition of danger sign, stock out of emergency medicines and inaccessible ambulance vehicles.

#### HCP absenteeism

Most participants moved to at least two HCs before finally coming to MRRH. HCP absenteeism at the initial primary HCs visited led to multiple referrals and delayed their access to BEMOC/CEMOC.

*“The labour ward door was open at the first HC we went to, but we did not find a nurse to help me. They should always remain at their work places. You should tell those at the HC that I went to that it is the main HC, but they mistreat patients, it’s a government health unit so they should work on people*.” (R4, referral with obstructed labour).

**Non-functional operating theatres** was a problem common to all the referring CEMOC centers during our study period. Other than the obstetric indication for referral, a client had an additional reason for transfer; the theater power generator had no fuel, so the caesarean section (C/S) instruments could not be sterilized due to power outages.

Absenteeism of doctors, midwives and anesthetic officers as seen contributed to the non-functionality of the operating theatres as reflected in the excerpt below

*“The health workers said that since I had already pushed, they should take me to Mbarara hospital directly, because they had not had electricity for two days and so didn’t have the equipment to use*.” (R11, primipara, referral with obstructed labour, over 200kms moved).

*“They could not operate on me; they said that the only doctor who could operate upon me had not worked that day*.” (R6, para 1 + 0, referral with obstructed labour from a HC IV, about 34kms away)..

#### Stock out of emergency medicines

Participants reported that the medicines to treat them were not available at the primary referral points so they were sent to MRRH to be able to access them.

*“I had high blood pressure and they said they wouldn’t manage me. They didn’t have my kind of drugs*.” (R3A referral with eclampsia, told part of her transfer story by her mother, R3B.)

#### Inaccessible ambulance vehicles

The referring health centers had ambulance vehicles but these were inaccessible because they were grounded, in poor mechanical condition or referrals lacked money to fuel them. Participants felt that health centers needed to have standby ambulance vehicles, ready to transport them to referral points.

*‘’The one for the health center had a technical problem, so we hired a vehicle from town. That further wasted our time moving up and down looking for a vehicle to bring us to the big hospital”* (R10, referral from a HC about 34kms away).

Irrational use of government ambulance vehicles was rampant. Participants who afforded to fuel H ambulance vehicles to transport them urgently to hospital, were disappointed when these were used to transport other normal passengers instead. They made many stop-overs dropping off these passengers before finally bringing them to Mbarara regional referral hospital.

*“We could have reached Mbarara town at 10 am but instead of dropping me first; me who was not well at all, instead the driver had many stop overs dropping off the other people who were not ill, imagine I was delayed for about 3 hours and you know what that means when one has pain. Remember my husband is the one who fueled that ambulance vehicle. Our complaints to this driver fell on deaf ears.”* (R2A referral with obstructed labor).

### Barriers to referral

The theme ‘barriers to referral’ emerged from the subthemes of; previous unpleasant referral experience, previous birth experience, poor ambulance system and long distance to referral center.

#### Poor ambulance service

Some of the transport means that they secured were rather risky and uncomfortable. Bad terrain where motor cycles don’t move easily hindered their transit. Some distance they would walk as the motor cycles were being pushed. Some journeys would be temporarily halted, as they waited by the roadside for their birth companions to look for vehicles for hire before they could resume. This caused further delays to reach the nearest CEMOC centers.

*“We couldn’t move any more due to the pain and being 3 people on one motor cycle, so we waited by the roadside as my husband went to look for a vehicle to transport us to Lyantonde, then from Lyantonde he had to hire another vehicle to bring us here.”* (R1 from Sembabule District, over 200kms moved to MRRH).

*“The vehicle with a pregnant woman.... uh.... and they would slow down where there were potholes and humps. And now from Sembabule to Lyantode....uh........ [Shaky voice and tears in her eyes...]”* (R9 referral with ruptured uterus secondary to obstructed labour, referred about 200kms from MRRH).

#### Previous birth experience

There was no perceived need to go to give birth at a HC for participants who have always had prior successful home births. These delayed to decide to seek care.

*I have never had strong labour pains, I usually see a gush of water then I push my babies safely from home. I have had two twin births at home. I hoped to have these successfully at home too. When the first baby came out I got pains again and because I have already had twins, I knew how the whole process goes. I usually give birth from my home, my garden my babies are always healthy not this business of moving from one HC to the other.”* (R10, referral with retained twin).

#### Previous unpleasant referral experience

Some participants who had heeded prior antenatal referral advice timely only to have poor outcomes with previous pregnancies did not see the essence of reporting to a HC early this time round. They stayed home and ended up as late self-referrals with obstructed labour and impending rupture of uterus with current pregnancy. This can be seen from the excerpt below;

*“I was told that the baby was not in a good position so when I was told to go to the main hospital when am due. I came here in time but there was no electricity at the time I was to be operated upon .By the time they operated me, they found that the baby had died inside my womb. So what did it help to come here early? You can still lose a baby even after reporting early to hospital*” (R8 referral with obstructed labor- transverse lie in advanced labor, who had a stillbirth previously referred from a HC III about 35kms away).

#### Financially draining

The cost of referral was overwhelming to most of the participants who had to borrow money or sell personal property to raise transport fare, fuel ambulance vehicles or hire vehicles and for upkeep during the long hospitalizations at MRRH. Having to go back home to solicit for transport fare worsened their delay and increased their risk for developing complications. Car hire costs were also hiked because car owners had to cater for car wash fees in case the woman soiled their car with blood or liquor amnii.

“*Getting a special hire taxi was not easy either, instead of 10.000 shillings (3 USD) they charged us 30.000 shillings (10 USD). Because we might soil their car, they had to charge extra money for car wash after the journey.”* (R9, referral with retained twin).

*“From Kabwohe to Kashaka it was 30000 and to Mbarara 70000.They said that it was high blood pressure, they didn’t have medicine for it, they told me to look for it, it went for 60,000 yet I had only 10000 on me .I had to send for a goat to be sold to raise money. Some drugs we have bought at 60000, 80000, or 100000. I thought drugs should be free since it’s a government hospital.”* (R3B, a 50-year-old mother to R3A referral with eclampsia.)

#### Long distance to referral center

The distance to Mbarara regional referral hospital was long. Participants worried about raising the fare and later on the upkeep at MRRH which is located in an urban area. They chose to seek care at nearby health centers yet these were not equipped to handle their conditions. This led to the need for multiple referral before coming to MRRH and subsequent second delays.

*“Well, I was told in the antenatal clinic at Bubaare that I have to come to MRRH for delivery, but when I pushed one of the babies and the second one was not coming, I thought to myself; let me get 1000 shillings and go to Kabwohe HC, there are also health care providers like those at Mbarara regional referral hospital where I need a lot of money to get there. Plus the upkeep money needed in that town is too much*” (R9, referral with retained twin, third time with multi-foetal gestation).

## Discussion

This study about experiences of obstetric referral yielded 4 interconnected themes and 19 sub-themes; poor client-HCP communication, poor quality of care, enablers and barriers to referral. Most women experienced delay, exhaustion, and poor quality of care at referral points. The referral process was a worrisome journey, financially draining, and women felt that they had been treated unfairly by health care providers. Individual socio-economic constraints and health system delays negatively influenced their experience of obstetric referral process.

Our participants experienced gross difficulty before finally accessing care at the main regional referral hospital in the region, MRRH. They underwent multiple referrals owing to the inability of the lower HCs to provide BEMOC and CEMOC. WHO advocates for improvements in timely emergency obstetric care (EMOC) which is care to women with obstetric complications and quality care if reduction in the unacceptably high global maternal and neonatal mortality and morbidity are to be reduced. In fact the Sustainable Development Goals (SDGs) agenda aims to reduce the average global MMR to less than 70 maternal deaths per 100,000 live births by 2030 [10, 28, 29].

However, Uganda’s maternal and newborn mortality remains high at 336 maternal deaths per 100,000 live births and 27 newborn deaths per 1,000 live births respectively and corresponds to ‘three delays’; delayed decision to seek care, delayed transportation and appropriate healthcare at HC. In Uganda 15 women die every day from pregnancy and childbirth-related causes, 94 babies are stillborn, and 81 newborn babies die. This translates into 695,701 deaths each year due to complications during pregnancy and childbirth [30, 31]. Addressing the 3 major delays associated with maternal and newborn deaths requires identifying barriers at each step of delivering care, strengthening the health system to increase access to emergency obstetrics [30, 32]. Our study findings indicate that Uganda is in dire need of efforts to expedite the achievement of the SDG 2030 maternal health goals.

Such efforts to improve obstetric referral include improvements in hospital supplies and trainings staff to consolidate teamwork and update emergency care skills. Trainings in customer care, emergency obstetric care drills have been found to be vital for performance improvements among obstetric health care providers [15, 33, 34].

Maintenance of equipment, charitable donations for equipment, drugs, supplies for blood donation and transfusions have been shown to greatly enhance health care providers’ efforts to provide emergency obstetric care [15, 35–38]. Some secondary reasons for referral (health system barriers) in our study included lack of health facility supplies. Systems to sustain such interventions for south western Uganda could improve maternal health services.

Health care provider absenteeism caused the third delay at the primary CEMOC health facilities. The single most important factor contributing to delays and associated adverse outcomes for mothers and babies in Uganda is the failure of doctors to be present at work during contracted hours [39, 40]. Women in our study failed to access timely CEMOC because maternity wards were closed or the doctor and anesthetist to carry out surgery were absent. We did not explore extensively the reasons for HCP absenteeism in the current study. However some studies suggest that staff attendance to duty could be boosted by providing housing facilities for them within the health center premises so that they are always in the vicinity to provide the desired emergency obstetric services when called upon [41].

The ordeal of finally reaching a HC and still end up with poor outcomes is disheartening and will discourage women from seeking skilled birth attendance .Antenatal care appears to facilitate utilization of emergency obstetric care especially for women with socio-demographic disadvantages. It is believed that women could take up referral advice if they attended antenatal care where they can understand why they are being referred [42]. On the contrary, antenatal referral advice of at-risk women was not heeded by some of our participants who preferred to give birth from home owing to previous unpleasant referral experiences. Poor HCP attitudes and third delays have potential to keep away from HCs because of no perceived benefit from attendance.

Following late referral, women in our study developed complications like puerperal sepsis and vesico vaginal fistula. Many women get sequelae after a near miss event; for every maternal death about 6 women suffer severe morbidities, some of which are lifelong. The odds of dying or getting a maternal morbidity are higher among those referred late from other HCs [43]. Although no deaths were reported during our study period, most participants suffered major morbidity like secondary infertility following hysterectomy in primiparous women. Our findings therefore further confirm the alarming morbidity trends in Uganda; that for every woman or girl who dies from a childbirth complication, an estimated 20 or 30 suffer injuries, infections or life-long disabilities including obstetric fistula [29].

Most participants in this study reported rude, cold communication and uunethical behavior from HCPs who solicited money from them in a government health facility where user- fees were abolished. Negative medical communication constitutes a major complaint about obstetric care services. Positive attitudes of health workers and provision of adequate medical information may promote a more positive hospital experience of women in need of obstetric care and enhance future attendance [44, 45].

Poor staff attitudes notwithstanding, our findings also point to delayed adequate decision making in clinical care where women in obstructed labor pushed for long at primary care points and were retained even after they requested for transfer. Pre-service curricula for HCPs in Benin, Malawi, Tanzania, and Uganda reveal gaps in women-centered care, inclusion of women in decision making and fundamental human rights of individuals [46] which ultimately do not conform to those recommended by the International Confederation of Midwives (ICM ) competence Framework .The skill, knowledge and behavior gaps for midwifery HCPs in Uganda need to be addressed [46].

In Kenya, inhospitable formal service providers hindered slum dwellers to seek emergency obstetric care services [47]. In our study, services were also sometimes not user-friendly owing to poor communication from the health care providers. Stakeholders suggest that there should be short competency-based in-service trainings in emergency obstetric care to improve knowledge/skills for better clinical practice and subsequent quality of care [48]. Perhaps trainings in proper execution of obstetric referral, customer/ respectful maternity care are desired now for our setting.

Delay to reach care seems to be the weakest link for all programs addressing the maternal morbidity and mortality question [49]. Warren also found that distance, cost to health facility, diversion to inappropriate health facilities are hindering factors to urgent access to receiving obstetric care [7, 50].

Health facilities have no capacity to adequately respond to and manage women with obstetric complications [51]. Studies in Ethiopia and northern Karnataka region of India investigating readiness of HC to manage eclampsia, found poor availability of dipsticks for proteinuria tests, caesarean sections, reported across almost all the public health facilities. Magnesium sulphate, the drug of choice for controlling convulsions was more available in private for-profit health facilities compared with public facilities [52, 53]. Participants in our study were also referred mostly from public health facilities due to stock- outs of magnesium sulphate. This can be devastating in the event that a health worker desires to urgently bring a convulsion to a halt.

Transport delays like no ambulance vehicles, ambulance vehicles with mechanical problems, no fuel for ambulances coupled with long distance to the main referral HC were rampant in our study. Such inadequacies also inflate the treatment costs Transportation of referred pregnant women to appropriate HCs and en route stabilizing care plays a pivotal role in preventing maternal deaths in low-income and middle-income countries [54].

Maternal deaths, stillbirths and neonatal deaths were common outcomes in India among women who requested to use the free ambulance services but failed. Strategies are required to reduce non-use of ambulance for women with obstetric emergencies [54]. Women who delayed accessing emergency obstetric care health facilities owing to transport delays in our study also had stillbirths and early neonatal deaths though luckily no maternal death occurred during our study period. Other than delaying at lower health units, delay also occurred at the Traditional birth attendants(TBAs) place. This echoes Kaye and colleagues’ findings in the study about lived experiences of having sustained a ruptured uterus where it was reported that many women experienced delays at home, at the TBAs or at the lower health clinics from where they were later referred or from where they referred themselves arriving too late to Mulago National Referral Hospital [55].

In a qualitative study done in three States of Australia of New South Wales, Victoria and Western Australia exploring experiences of having survived a severe postpartum haemorrhage and an emergency hysterectomy, women described their experience as being between life and death [56]. Women in the current study were deeply worried, death seemed imminent as they observed a colleague die after an eclamptic fit, some felt so ill during transfer to MRRH and were not certain they could make it.

Overall, challenges to obstetric referral are due to both the client and external factors. External environment factors according to the Andersen’s Health care utilization model can be equated to the barriers and health care system inadequacies that caused delays in referral. The predisposing factors relate to the client background characteristics like poor social economic status while the need factors were the actual direct indications for referral.

Our findings are similar to those of studies from Pakistan, Bangladesh and sub-Saharan Africa. In Pakistan, diverse factors limit women’s access to Emergency Obstetric Care (EmOC) services. EmOC services were unavailable in most health facilities due to staff absenteeism, geographic remoteness, delayed access, and ambulance shortages all of which jeopardize the transfer of seriously ill clients to higher level care facilities. [57]. Women in the current study experienced similar barriers as they sought EMOC. In Bangladesh, women needing emergency obstetric care services needed emergency referral, and either need to walk to a transport point or be carried in makeshift stretchers, resulting in much physical and mental distress [58]. Women in the current study utilized motor cycle transport sometimes with multiple passengers on a motor cycle which was rather uncomfortable. One required immense resilience to use such means of transport on rough roads through hilly terrains.

In Mozambique and Tanzania challenges to accessing emergency obstetric care also point to a weak referral process due to its limited functionality [59]. Non-functional referral systems linked to high maternal mortality ratios are common mainly with LMIC countries of the world; Countries in Sub-Saharan Africa and some from Asia. Data on obstetric referral in high income countries is scanty. This could be that their referral systems are efficient.

However, experiences from near-miss events in both high income and countries are similar. The short-term and long-term emotional and physical devastation involved is the same regardless of socio-economic status. Partners of survivors of severe life-threatening pregnancy complications (near-miss) in the UK, experienced powerlessness and exclusion. Witnessing the emergency was shocking and distressing. The long-term emotional effects for some were profound; some experienced depression, flashbacks and post-traumatic stress disorder months and years after the emergency. These, in turn, affected the whole family. They coped with support from family or staff and clear, honest communication from medical staff [60]. On the contrary, staff communication to our participants as seen was largely unfriendly. Our study however never explored the coping strategies of the spouses and significant others.

Challenges with a poor obstetric referral system in south western Uganda are not only limited to women. Health care provider perspectives of the obstetric referral system in South Western Uganda revealed several constraints that range from inconsistencies of ambulance and anesthesia services, electric power, medical supplies, no communication prior to referral, support supervision, harassment by colleagues and no feedback from MRRH to lower health facilities [61]. These HCPs suggest that staffing levels should be increased .This could address the problem of HCP absenteeism in the event that some health centers are closed to service provision when their only staff is off duty for whatever reasons. Increasing the resources at the health provider level is important in achieving international targets for maternal and neonatal health outcomes and for bridging inequities in access to essential maternal and newborn healthcare [62] .

### Strengths of the study

The study documented views of the individuals that interface with the health care system themselves. This first-hand information is important to policy influencers for maternal health services. Interviews were conducted shortly after stabilization while the respondents were still in hospital. This ensured that the near-miss experiences were still fresh in their memory.

Most studies around the obstetric referral explored the views of health care workers. Where women have been are involved, studies are usually describing care at the final referral destinations, not experience along the entire referral pathway or have been done elsewhere. The current study adds the women’s perspective in rural South Western Uganda to literature.

### Limitations of the study

Interviewing these women when they were still hospitalized with fresh memory of some traumatizing events like loss of babies to birth asphyxia, loss of wombs to hysterectomy and development of vesico-vaginal fistula led to emotional breakdown. Interviews for those women who got emotional breakdown were rescheduled or completed by their support persons in the case of those with eclampsia and could not narrate their ordeal after onset of a status eclampticus.

The number of women interviewed from a few referring health centers was relatively small to give comparable results. We hope that duplication of this study with more participants and referral centers could produce more comparable results.

### Study implications

Overall, our study reveals rampant negative experiences for women along the obstetric referral pathway for rural south western Uganda due to poor socioeconomic status of women in labour and health system organizational discord. This will derail the country efforts at achieving sustainable development goal 3 (SDG 3); achieving good health and wellbeing for everyone, including good maternal health. WHO advocates for positive postpartum experiences but these cannot be realized with the physical and emotional morbidity experienced following these near-miss events.

## Conclusions

In conclusion, our study has revealed that the numerous individual, community and health system barriers to referral aggravated the negative experience of having an obstetric emergency and later being referred for care. Participants experienced delay in receiving emergency obstetric care, poor quality of care, the process was financially draining and they developed complications the warranted prolonged hospitalization. Women’s interface with the current obstetric referral pathway in southwestern Uganda reveals that the latter is wanting. A prior study shows that health care providers are equally constrained by the health care system. Multi-sectoral concerted efforts will be handy to expedite improvements in emergency obstetric referral for western Uganda by addressing the prevalent multifaceted challenges.

## Recommendations

From the narratives of these participants, it can be deduced that service improvements which address user-views are important to impact on health seeking behavior and utilization. The health care system needs to focus on improvements in obstetric referral pathways so as to ensure a better continuum of care from the communities to health facilities.

Nurses and midwives need to adhere to the ethical code of nursing conduct, improving provider-client communication and relationship in order to heal the tainted nursing image and improve public opinion. This will increase demand for nursing services and skilled obstetric care in particular. Labour care guide use needs to be emphasized so that labour complications can be recognised early for timely referral since some of the participants developed complications like obstructed labour while already at health facilities. Job aids and tools proper execution of obstetric referral should be develop and disseminated.

The ministry of health should improve the staffing norms at health facilities so that there are enough doctors and midwives to run the health units when some are off duty for whatever reasons to address the problem of absenteeism.

Consistent logistical support in form of emergency drug stocks (Magnesium sulphate), funds to fuel HC electric power generators, sterilisation of instruments and fuel for ambulances is suggested for lower HCs so that multiple client transfers and third delays are mitigated.

The available operating theatres at lower health facilities if equipped and are more functional will save women multiple referrals.

Birth planning and emergency preparedness campaigns should be mounted for pregnant women so that they are guided to seek timely care to avoid delays.

Factors responsible for health care provider absenteeism in south western Uganda should be explored.

Training healthcare providers in respectful maternity care may improve both quality of care and positive patient experiences. HCPs may benefit from refresher sessions on customer care, teamwork and proper procedures for execution of obstetric referral.

An implementation study to explore strategizes to improve the obstetric referral pathways for rural south western Uganda and parts of central Uganda is suggested.

## Electronic supplementary material

Below is the link to the electronic supplementary material.

Supplementary Material 1

## Data Availability

These are available from corresponding author upon reasonable request and with approval from the Research Ethics Committee.

## Abbreviations

ADHO: Assistant District Health Officer
ANC: Antenatal Clinic/care
HC: Health Center
HCP: Health Care Provider
MRRH: Mbarara Regional Referral Hospital
VHT Member: Village Health Team Member.
WHO: World Health Organization
UNCST: Uganda National Council for Science and Technology
