# Supplementary material for: Experiences of referral with an obstetric emergency: voices of women admitted at Mbarara Regional Referral Hospital, South Western Uganda
Source: BMC Pregnancy Childbirth. 2023 Jul 6;23:498. doi: 10.1186/s12884-023-05795-z (PMC10327367; doi:10.1186/s12884-023-05795-z)
Supplement: Supplementary file 1 — Supplementary Material 1 [file 12884_2023_5795_MOESM1_ESM.pdf]

INTERVIEW GUIDE FOR WOMEN REFERRED WITH OBSTETRIC COMPLICATIONS TO MRRH

I am Ms. **Harriet Nabulo** conducting a study about the experiences of women referred with obstetriccomplications to Mbarara regional referral Hospital. Since you were sent here for care, am goingto ask you to narrate to me what you went through before finally arriving here. This interview will take about 45 minutes of your time.

Participant code: …………………………………………………………………………………………………

Initials of Interviewer: …………………………………………………………………………………………...

Date of Interview: ……………………………………………………………………………….

S/N	ISSUE TO BE EXPLORED	QUESTIONS
1	Demographic characteristics	Please tell me about yourself; initials, age , parity, village, religion, marital status, address, level of education, initials of next of kin and your relationship with next of kin
2	Experiences encountered during the transfer process	Please tell me about what you have experienced<br>Why were you sent here?<br>Who sent you here?<br>How were your interactions with the health care workers and other staff?<br>How did you find the care you received before coming here?<br>How was your journey to this hospital?
3	Enablers to referral	What facilitated your transfer journey to this hospital? Transport means, Cost of transport, birth companion, escort, distance to this hospital, kind of support<br>How long was the journey?
4	Barriers to referral	What did not go so well during your transfer journey?<br>What was the approximate distance to this hospital,

		What difficulties did you face as you transferred here?<br>Please share anything you wish about this transfer experience.<br>What can be done better?

The end. Thank you.
